# Characterization of Obesity Phenotypes in *Psammomys Obesus* (Israeli Sand Rats)

**DOI:** 10.1155/EDR.2000.177

**Published:** 2000

**Authors:** Ken R. Walder, Richard P. Fahey, Greg J. Morton, Paul Z. Zimmet, Greg R. Collier

**Affiliations:** 1 Metabolic Research Unit School of Health Sciences Deakin University Waurn Ponds Victoria 3217 Australia; 2 lnternational Diabetes Institute Caulfield Victoria 3162 Australia

## Abstract

*Psammomys obesus* (the Israeli sand rat) has been well studied as an animal model of Type 2 diabetes. However, obesity phenotypes in these animals have not been fully characterized. We analyzed phenotypic data including body weight, percentage body fat, blood glucose and plasma insulin concentration for over 600 animals from the *Psammomys obesus* colony at Deakin University to investigate the relationships between body fat, body weight and Type 2 diabetes using regression analysis and general linear modelling. The body weight distribution in *Psammomys obesus* approximates a normal distribution and closely resembles that observed in human populations. Animals above the 75th percentile for body weight had increased body fat content and a greater risk of developing diabetes. Increased visceral fat content .was also associated with elevated blood glucose and plasma insulin concentrations in these animals. A familial effect was also demonstrated in *Psammomys obesus*, and accounted for 51% of the variation in body weight, and 23–26% of the variation in blood glucose and plasma insulin concentrations in these animals. *Psammomys obesus* represents an excellent animal model of.obesity and Type 2 diabetes that exhibits a phenotypic pattern closely resembling that observed in human population studies. The obesity described in these animals was familial in nature and was significantly associated with Type 2 diabetes.


*Int. Jnl. Experimental Diab. Res.*, Vol. 1, pp. 177–184
Reprints available directly from the publisher
Photocopying permitted by license only

© 2000 OPA (Overseas Publishers Association) N.V.
Published by license under
the Harwood Academic Publishers imprint,
part of The Gordon and Breach Publishing Group.
Printed in Malaysia.

### Characterization of Obesity Phenotypes in *Psammomys Obesus* (Israeli Sand Rats)

KEN R. WALDER[a,*], RICHARD P. FAHEY[a], GREG J. MORTON[a], PAUL Z. ZIMMET[b] and GREG R. COLLIER[a]

[a]*Metabolic Research Unit, School of Health Sciences, Deakin University, Waurn Ponds 3217, Victoria, Australia;* [b]*International Diabetes Institute, Caulfield 3162, Victoria, Australia*

*(Received in final form 28 February 2000)*

**_Psammomys obesus_ (the Israeli sand rat) has been well studied as an animal model of Type 2 diabetes. However, obesity phenotypes in these animals have not been fully characterized. We analyzed phenotypic data including body weight, percentage body fat, blood glucose and plasma insulin concentration for over 600 animals from the _Psammomys obesus_ colony at Deakin University to investigate the relationships between body fat, body weight and Type 2 diabetes using regression analysis and general linear modelling. The body weight distribution in _Psammomys obesus_ approximates a normal distribution and closely resembles that observed in human populations. Animals above the 75th percentile for body weight had increased body fat content and a greater risk of developing diabetes. Increased visceral fat content was also associated with elevated blood glucose and plasma insulin concentrations in these animals. A familial effect was also demonstrated in _Psammomys obesus_, and accounted for 51% of the variation in body weight, and 23–26% of the variation in blood glucose and plasma insulin concentrations in these animals. _Psammomys obesus_ represents an excellent animal model of obesity and Type 2 diabetes that exhibits a phenotypic pattern closely resembling that observed in human population studies. The obesity described in these animals was familial in nature and was significantly associated with Type 2 diabetes.**

*Keywords*: *Psammomys obesus*; Obesity; Type 2 diabetes; Body fat content; Familial

### INTRODUCTION

Obesity may be defined as a pathological increase in body fat content, and is a result of a sustained imbalance between energy intake and energy expenditure. It is an extremely common metabolic disorder, affecting approximately 17% of adult Australians [1] and 23% of adult Americans [2] and is epidemic in some developing countries. [3] Obesity is an extremely serious public health problem as it increases the risk of co-morbidities such as cardiovascular diseases, Type 2 diabetes, hypertension, musculo-skeletal disorders and certain cancers. [4,5] The commonest form of Type 2 diabetes, which may be defined as a pathological increase in blood glucose concentration, characteristically develops in obese, middle-aged individuals and, if not adequately controlled, can be extremely

*Corresponding author. Tel.: +61-3-52272883, Fax: +61-3-52272170, e-mail: walder@deakin.edu.au debilitating with further complications including blindness, renal failure and peripheral vascular insufficiency. Like obesity, Type 2 diabetes is highly prevalent in both affluent and developing societies. In the latter situation, it is now seen in younger age groups than in Europid communities and even in adolescence.[6] The reports of a spectacularly high prevalence of NIDDM of over 40% in adult Pima Indians of Arizona[7] and Micronesian Nauruans,[8] have been followed by other reports including prevalences in the Pacific Islands of Kiribati and Western Samoa of 11% and 16%, respectively, and a prevalence of 37% in the Melanesians of Papua New Guinea.[9] At the other end of the scale, very low prevalences of 1 to 2% have been found in parts of Africa and China. In Europid subjects, while there are only a few studies, they indicate rates around 5% in adult Australians[10] and Americans.[11]

Research into obesity and Type 2 diabetes has been much enhanced by the identification of animal models of these diseases. An excellent example of the insights into both normal and pathological processes gained from these models is that of leptin.[12] However, the most utilized animal models, including *ob/ob* and *db/db* mice and *fa/fa* rats, are single-gene models and may not closely represent the processes involved in the pathogenesis of human obesity and diabetes. This is because these complex diseases are generally thought to be polygenic or oligogenic in humans, with only rare and extreme cases due to single-gene mutations.

For these reasons the development and characterization of polygenic animal models of obesity and diabetes, which are likely to more closely reflect the pathological processes in human disease, are of considerable value to this field of research. Primates are thought to provide a very good model of human obesity and diabetes. However the drawback with these animals is the difficulty associated with handling and housing the animals, and the long lifespan, which complicates longitudinal studies.

*Psammomys obesus* (the Israeli sand rat) is a unique rodent model of obesity, insulin resistance and Type 2 diabetes. Its natural habitat is the desert regions of the Middle East, where it subsists on a diet of saltbush and remains lean and normoglycemic.[13] However, when housed in laboratory conditions and fed *ad libitum* chow, a diet on which many other rodent species remain healthy, a range of metabolic responses have been observed.[14,15] By 16 weeks of age approximately one third of the animals have normal glucose tolerance, one third are hyperinsulinemic and normoglycemic, and one third develop diabetes.[15] The stages of diabetes development have been characterized and defined in *Psammomys obesus*, and studies are ongoing to investigate the physiological processes involved. *Psammomys obesus* have hepatic insulin resistance even before the development of hyperglycemia and hyperinsulinemia,[16] and have been shown to have a defective insulin receptor-signaling pathway.[17,18] Hyperproinsulinemia, reduced pancreatic insulin storage capacity and beta-cell apoptosis have also been demonstrated in these animals.[19,20] Other phenotypes noted include dyslipidemia and hyperphagia.[15,21] The relationship of blood glucose and plasma insulin concentrations forms a continuous curve in *Psammomys obesus* identical to "Starling's curve of the pancreas" in human populations.[22,23] This heterogeneous response indicates that *Psammomys obesus* probably represents a polygenic animal model of human diabetes.

*Psammomys obesus* also exhibit a wide range of body weight, and may represent a polygenic animal model of obesity resembling the disease in human populations. However, the development of obesity and characterization of body weight and body fat distribution have not been well defined. In this study we analyzed data from the *Psammomys obesus* colony at Deakin University in Geelong, Australia to investigate the obese phenotype in these animals, and in particular to define obesity and its relationship with Type 2 diabetes.

### RESEARCH METHODS AND PROCEDURES

A colony of *Psammomys obesus* is maintained at Deakin University, Geelong, Australia. Breeding pairs are fed *ad libitum* a diet of lucerne and chow, and are maintained using the San Poiley outbreeding method. Data included in this study were from animals from the 12th to the 16th filial generations. Experimental animals were weaned at 4 weeks of age and given a diet of standard laboratory chow, from which 12% of energy was derived from fat, 63% from carbohydrate and 25% from protein (Barastoc, Pakenham, Australia). Animals were housed in a temperature-controlled room (22 ± 1°C) with a 12–12 h light-dark cycle (light 06:00–18:00, dark 18:00–06:00).

This study included data from 633 animals from the colony for which phenotypic data was available at 16 weeks of age, prior to being included in experiments. Whole blood glucose was measured using an enzymatic glucose analyzer (Model 27, Yellow Springs Instruments, Ohio). Plasma insulin concentrations were determined using a double-antibody solid phase radioimmunoassay (Phadeseph, Kabi Pharmacia Diagnostics, Sweden). The antibody in this kit does not differentiate between insulin and proinsulin, so some of the insulin measurement may actually be proinsulin.

Estimates of body fat content in *Psammomys obesus* were made by surgically removing and weighing selected major adipose tissue depots from animals after sacrifice. These fat depots included the perirenal, suprascapular (includes both white and brown adipose tissue), mesenteric and intramuscular (from between the heads of gastrocnemius). The combined weight of these fat pads was expressed as a percentage of body weight, and was taken to be an estimate of percentage body fat in these animals (%fat).

All animals were maintained in accordance with the Code of Practice of the National Health and Medical Research Council of Australia, and all procedures were carried out subject to the approval of the Deakin University Animal Experimentation Ethics Committee.

All data are expressed as mean ± standard deviation. Distribution of data was tested using Kolmogorov–Smirnov test for normality, and data was normalized by transformation if required for further analysis. Group means were tested by one-way ANOVA with a *post hoc* LSD test, and relationships between variables were tested using either linear regression analysis or general linear model testing. Results were considered significant at $p < 0.05$.

### RESULTS

### Definition and Characterization of Obesity in *Psammomys Obesus*

*Psammomys obesus* from the Deakin University colony exhibit a wide range of body weight. Figure 1 shows a frequency histogram for the body weight variable in a total of 633 animals. The mean body weight for the colony at 16 weeks of age was 198.7 ± 30.9 g, with a range from 109–276 g. Male animals were significantly heavier than females (211.7 ± 26.0 *vs.*

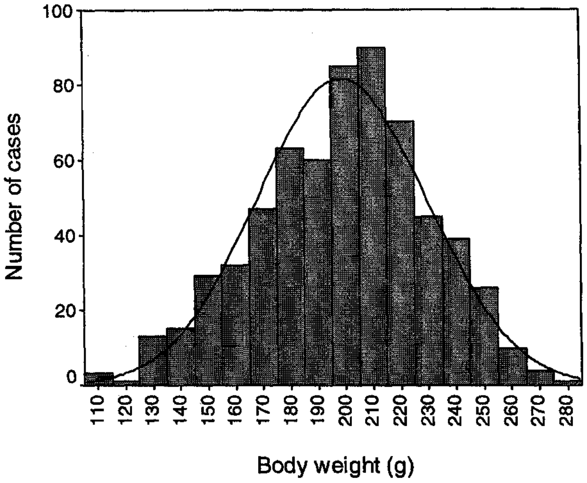

FIGURE 1 Frequency distribution of body weight at 16-weeks of age in *Psammomys obesus*.

177.2 ± 25.9 g; $p < 0.001$), and the ranges were similar (male: 133–276; female: 109–238).

To define obesity in *Psammomys obesus*, it would be most appropriate to determine a cut-off point above which the risk of metabolic complications is increased. We have collected a large amount of data pertaining to the development of Type 2 diabetes in these animals. A summary of the distribution into phenotypic groups at 16 weeks of age is given in Table I.

We have documented a progressive increase in the body weight of animals from Group A through to Group C. The body weights of animals in these groups are shown in Figure 2. For both sexes, diabetic (Groups C and D) animals were significantly heavier than Group B, which in turn were heavier than Group A ($p < 0.001$ by ANOVA). These data suggest that excessive body weight is related to the development of insulin resistance and diabetes in *Psammomys obesus*. Regression analysis using general linear modeling confirmed that 20% of the variation in blood glucose concentration (after adjustment for gender and plasma insulin concentration) could be explained by variation in body weight in these animals.

The risk ratio of developing diabetes was investigated in the heaviest animals in the colony. For each sex, at 16 weeks of age, those animals over the 75th percentile of the body weight distribution had increased risk of diabetes. In males, the 75th percentile was 230 g and animals over this body weight had a 2.3-fold increased risk, while females over the 75th percentile (196 g) had a 2.9-fold increased risk compared with the general *Psammomys obesus* population. Therefore, it seems plausible to define obesity in *Psammomys obesus* as a body weight at 16 weeks of age of greater than 230 g for males, and greater than 196 g for females.

In addition to showing a metabolic risk when defining obesity, it is obviously important to demonstrate an increased body fat content in obese animals. We have also investigated body

TABLE I Distribution of 16-week old *Psammomys obesus* into stages of diabetes development

| Group* | Male | Female | Total |
|---|---|---|---|
| A: normoglycemic, normoinsulinemic | 127(38%) | 100(48%) | 227(42%) |
| B: normoglycemic, hyperinsulinemic | 115(35%) | 85(40%) | 200(37%) |
| C: hyperglycemic, hyperinsulinemic | 82(25%) | 21(10%) | 103(19%) |
| D: hyperglycemic, normoinsulinemic | 7(2%) | 5(2%) | 12(2%) |

*Hyperglycemic = blood glucose > 8.0 mmol/L; hyperinsulinemic = plasma insulin > 150μU/ml.

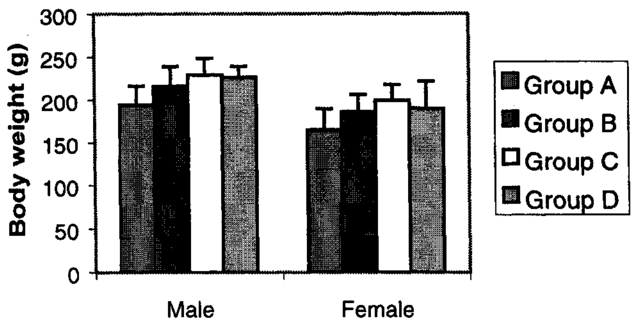

FIGURE 2 Body weight (mean ± s.d.) of *Psammomys obesus* from each stage of diabetes development. Group A animals were normoglycemic and normoinsulinemic, Group B were normoglycemic and hyperinsulinemic, Group C were hyperglycemic and hyperinsulinemic, and Group D were hyperglycemic and hypoinsulinemic. *For both sexes, $p < 0.001$ by ANOVA.

fat content and distribution in *Psammomys obesus* by dissection of the major fat depots after sacrifice of the animals. The fat depots excised and weighed include the perirenal, suprascapular, mesenteric and intramuscular (from between the heads of gastrocnemius). The weights of each of these individual depots closely correlated with the body weight of the animal. Table II lists the partial correlation coefficients (controlling for sex) and the significance level for each of these fat depots with body weight.

The combined fat pad weight, expressed as a percentage of total body weight, is normally distributed in these animals and has been used as an estimate of percentage body fat in *Psammomys obesus*. This variable was highly correlated with body weight in these animals ($r = 0.63$, $p < 0.001$; Fig. 3), although this relationship is clearly heterogeneous as evidenced by the variation around the regression lines in Figure 3. In the Deakin University colony, estimated percentage body fat (%fat) was significantly increased in Group C (diabetic) animals compared to Group B (hyperinsulinemic) animals, which in turn had greater %fat than Group A animals (Fig. 4; $p < 0.001$ by ANOVA).

If *Psammomys obesus* were defined as "obese" as described above (over the 75th percentile), and animals were defined as "lean" below the 25th percentile, the %fat for the groups is shown in Figure 5. As expected, in both males and females the %fat was significantly greater in obese compared to lean animals (Fig. 5; $p < 0.001$ by ANOVA).

TABLE II Partial correlation coefficients (adjusted for gender) for various fat depots and body weight in *Psammomys obesus*

| Fat depot | Correlation coefficient | $p$-value |
|---|---|---|
| Perirenal | 0.42 | < 0.001 |
| Suprascapular | 0.50 | < 0.001 |
| Mesenteric | 0.39 | < 0.001 |
| Intramuscular | 0.48 | < 0.001 |

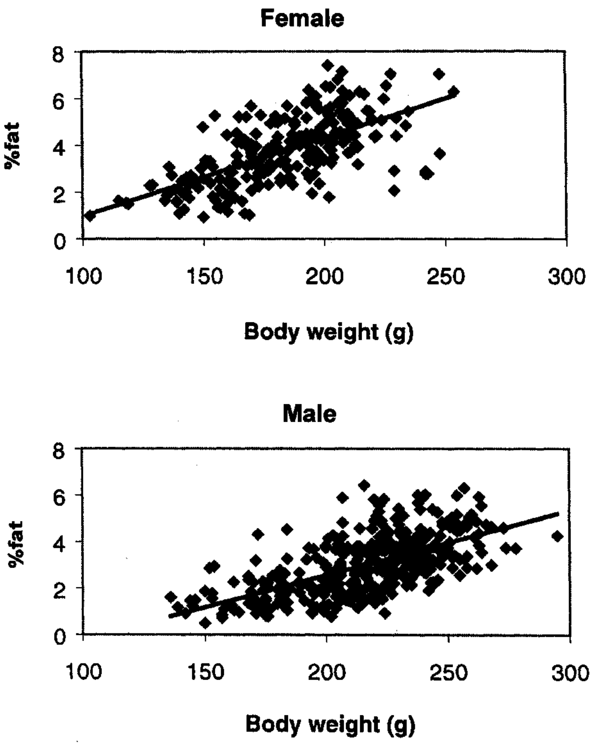

FIGURE 3 Correlation between %fat and body weight in female (upper panel) and male (lower panel) *Psammomys obesus*. For females, $n = 235$, $r = 0.63$, $p < 0.001$; for males, $n = 380$, $r = 0.63$, $p < 0.001$.

Linear regression analysis was used to investigate the relationship between %fat and the individual fat depot weights and diabetes in *Psammomys obesus*. The blood glucose concentration, adjusted for sex, body weight and plasma insulin, was significantly associated with %fat ($p = 0.001$), and also independently with the weight of the perirenal ($p < 0.001$), mesenteric ($p = 0.001$) and intramuscular ($p = 0.006$) fat depots, but not with the suprascapular fat depot weight. Plasma insulin concentration (adjusted for sex, body weight and blood glucose) was also significantly associated with %fat ($p < 0.001$), and with perirenal ($p = 0.003$), mesenteric ($p < 0.001$) and suprascapular ($p < 0.001$) fat depot weights, but not with that of the intramuscular fat pad.

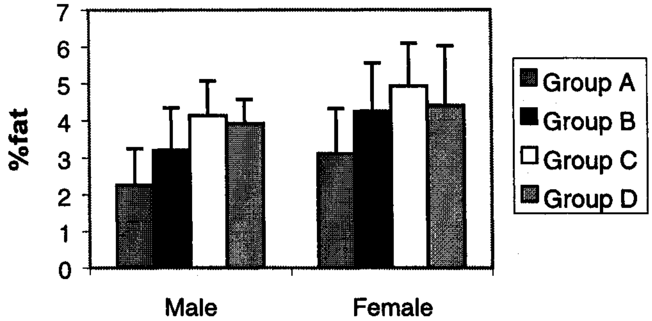

FIGURE 4 %fat of *Psammomys obesus* from each stage of diabetes development. Group A animals were normoglycemic and normoinsulinemic, Group B were normoglycemic and hyperinsulinemic, Group C were hyperglycemic and hyperinsulinemic, and Group D were hyperglycemic and hypoinsulinemic. *For both sexes, $p < 0.001$ by ANOVA.

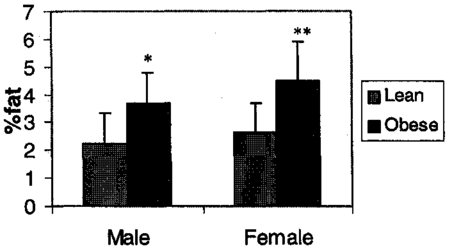

FIGURE 5 %fat of lean and obese *Psammomys obesus*. * Significantly greater than lean male ($p < 0.001$); ** Significantly greater than lean female ($p < 0.001$).

A summary of the phenotypic characteristics of animals classified as lean or obese is given in Table III. Within both sexes, the obese animals were significantly heavier, hyperglycemic, hyperinsulinemic and fatter than the lean group ($p < 0.001$ in all cases, by ANOVA).

### Familiality of Obesity Phenotypes in *Psammomys Obesus*

To address the question of the contribution of genetic influences to the development of obesity in *Psammomys obesus*, we used regression analyses with general linear models to assess the contribution of family membership to various phenotypic traits. The data included in the analysis was from a total of 613 animals comprising 166 families. A dummy variable pertaining to family membership was generated and assigned to each animal, and this variable was included in subsequent regression analyses. The number of siblings per family varied from 1 to 15, with a mean of $3.7 \pm 3.0$ and a median of 3. All variables were adjusted for sex prior to analysis.

Table IV lists the estimated genetic influences on each of the various phenotypic traits analyzed (calculated from the adjusted regression coefficients) along with the *F*- and *p*-values from the analysis. The familial effect on body weight was calculated to be 51%, while the effect on blood glucose and plasma insulin concentration were 23% and 26%, respectively. The genetic contribution to the weights of the individual fat depots was also calculated, and found to vary between 22 and 31%.

TABLE III Phenotypic characteristics of lean and obese *Psammomys obesus*

| | Male | | Female | |
|---|---|---|---|---|
| | Lean | Obese | Lean | Obese |
| Body weight (g) | 178 ± 14 | 244 ± 11 | 144 ± 12 | 209 ± 10 |
| Blood glucose (mmol/L) | 4.6 ± 2.1 | 9.9 ± 5.2 | 4.3 ± 0.9 | 7.3 ± 4.5 |
| Plasma insulin (μU/ml) | 160 ± 335 | 456 ± 273 | 86 ± 89 | 376 ± 273 |
| % fat | 2.2 ± 1.1 | 3.7 ± 1.1 | 2.6 ± 1.1 | 4.5 ± 1.4 |

TABLE IV Familial contribution to phenotypic characteristics of *Psammomys obesus*

| | Family effect | *F*-value | *p*-value |
|---|---|---|---|
| Body weight | 51% | 2.3 | < 0.001 |
| %fat | 32% | 2.3 | < 0.001 |
| Blood glucose | 23% | 1.8 | < 0.001 |
| Plasma insulin | 26% | 2.0 | < 0.001 |

### DISCUSSION

*Psammomys obesus* exhibit a wide range of body weight and body fat content in a laboratory setting. The distribution of body weight resembles that in human populations, suggesting that *Psammomys obesus* is an excellent rodent model of human obesity. There is no evidence of a bimodal distribution, which would be expected in a single-gene model.

Human obesity is defined based on large-scale population-based studies in which morbidity and mortality have been related to body weight or BMI (body mass index). The definition of obesity refers to a condition whereby the risk of adverse health outcomes is increased over a given BMI, relative body weight or body fat content.[3,24–26] We have defined obesity in *Psammomys obesus* by analyzing the relationships between body weight, body fat content and diabetes. We are unable to base this definition on overall mortality or other obesity co-morbidities, as these data are not available for this animal. Using the 75th body weight percentile as a guide, we propose that obesity be defined as a body weight at 16 weeks of age of greater than 230 g for male, and greater than 196 g for female *Psammomys obesus*. Animals above these arbitrary cut-off values for body weight have a significantly increased risk of developing diabetes as well as an elevated body fat content.

The contribution of body fat, both visceral and subcutaneous, to diabetes phenotypes was also investigated in *Psammomys obesus* using linear regression. Adjusted blood glucose and plasma insulin concentrations were significantly associated with the estimated percentage body fat (%fat) of the animal. Furthermore, the weights of the perirenal and mesenteric fat depots were also significantly and independently associated with both blood glucose and plasma insulin concentrations. These are both visceral adipose tissue stores, and suggest that *Psammomys obesus* may also represent a model of visceral obesity. In humans, visceral adipose tissue carries a higher risk of developing diseases such as diabetes, as well as a higher mortality independent of obesity *per se.*[27,28] Our findings in *Psammomys obesus* suggest a relationship between visceral adipose tissue and insulin resistance, which needs to be further investigated.

*Psammomys obesus* exhibit a wide range of phenotypic responses when removed from their natural environment and maintained under laboratory conditions.[13,14] The heterogeneity of the phenotypes observed under identical environmental conditions suggests a role for genetic factors in the disease processes. However, few studies have attempted to characterize the role of genetic factors in the disease process in these animals.[29] We used general linear modeling to analyze the variation within and between families of *Psammomys obesus* to estimate the contribution of genetic factors to various phenotypic traits (Tab. IV). Our results suggest that membership in a given family accounts for 23–26% of the variation in adjusted blood glucose and plasma insulin concentrations, and approximately half of the variation in body weight after adjusting for sex. The familial effect observed strongly supports the hypothesis that part of the heterogeneous variation in obesity and diabetes phenotypes in *Psammomys obesus* is due to genetic factors.

The findings of this study add weight to the suggestion that *Psammomys obesus* represents an excellent animal model of human obesity and Type 2 diabetes that, like humans, is likely to be polygenic in nature. Detailed phenotypic characterization of obesity and its relationship to diabetes in these animals should facilitate physiological and genetic studies seeking to determine the factors involved in both the etiology and pathology of abnormal energy balance and carbohydrate metabolism in *Psammomys obesus*. In addition, we also suggest that these animals provide a good model to investigate the role of visceral adipose tissue in the development of insulin resistance and diabetes.

### Acknowledgements

The authors would like to thank Maree McGlynn and staff at the Deakin University Animal House for maintaining the *Psammomys obesus* colony.

### References

[1] http://www.statistics.gov.au
[2] Flegal, K. M., Carroll, M. D., Kuczmarski, R. J. and Johnson, C. L. (1998). Overweight and obesity in the United States: Prevalence and trends, 1960-1994, *Int. J. Obesity*, **22**, 39-47.
[3] World Health Organisation, *Obesity: Preventing and Managing the Global Epidemic – Report of a WHO Consultation on Obesity, Geneva*, 3-5 June, 1997, Geneva: WHO.
[4] Bouchard, C. (1994). *The genetics of obesity*, Boca Raton: CRC Press.
[5] Zimmet, P. Z. and Collier, G. R. (1996). Of mice and (wo)men: The obesity (*ob*) gene, its product, leptin, and obesity, *Med. J. Aust.*, **164**, 393-394.
[6] Rosenbloom, A. L., Joe, J. R., Young, R. S. and Winter, W. E. (1999). Emerging epidemic of Type 2 diabetes in youth, *Diabetes Care*, **22**, 345-354.
[7] Knowler, W. C., Bennett, P. H., Hamman, R. F. and Miller, M. (1978). Diabetes incidence and prevalence in Pima Indians: A 19-fold greater incidence than in Rochester, Minnesota, *Am. J. Epidemiol.*, **108**, 497-505.
[8] Zimmet, P., Dowse, G., Finch, C., Serjeantson, S. and King, H. (1990). The epidemiology and natural history of NIDDM – lessons from the South Pacific, *Diabetes Metab. Rev.*, **6**, 91-124.
[9] Dowse, G. K., Hodge, A. M. and Zimmet, P. Z. (1996). Paradise lost: Obesity and diabetes in Pacific and Indian ocean populations. In: *Progress in Obesity Research*: 7, Angel, A., Anderson, H., Bouchard, C., Lau, D., Leiter L. and Mendelson R. (Eds.), London: John Libbey and Company.
[10] McCarty, C. A., McCarty, D. J., Van Newkirk, M. and Taylor, H. R. (1999). Self-reported diabetes and distribution of HbA1c in a population-based sample in Victoria, *Med. J. Aust.*, **170**, 238-239.
[11] Harris, M. I., Flegal, K. M., Cowie, C. C., Eberhardt, M. S., Goldstein, D. E., Little, R. R., Wiedmeyer, H. M. and Byrd-Holt, D. D. (1998). Prevalence of diabetes, impaired fasting glucose, and impaired glucose tolerance in U.S. adults. The Third National Health and Nutrition Examination Survey, 1988-1994, *Diabetes Care*, **21**, 518-524.
[12] Zhang, Y., Proenca, M., Maffei, M., Barone, M., Leopold, L. and Friedman, J. M. (1994). Positional cloning of the mouse obese gene and its human homologue, *Nature*, **372**, 425-432.
[13] Shafrir, E. and Gutman, A. (1993). *Psammomys obesus* of the Jerusalem colony: A model for nutritionally induced, non-insulin-dependent diabetes, *J. Basic Clin. Physiol. Pharm.*, **4**, 83-99.
[14] Kalderon, B., Gutman, A., Levy, E., Shafrir, E. and Adler, J. H. (1996). Characterization of stages in the development of obesity-diabetes syndrome in sand rat (*Psammomys obesus*), *Diabetes*, **6**, 717-724.
[15] Barnett, M., Collier, G. R., Collier, F. M., Zimmet, P. and O'Dea, K. (1994). A cross-sectional and short-term longitudinal characterization of NIDDM in *Psammomys obesus*, *Diabetologia*, **37**, 671-676.
[16] Ziv, E., Kalman, R., Hershkop, K., Barash, V., Shafrir, E. and Bar-On, H. (1996). Insulin resistance in the NIDDM model *Psammomys obesus* in the normoglycemic, normoinsulinemic state, *Diabetologia*, **39**, 1269-1275.
[17] Kanety, H., Moshe, S., Shafrir, E., Lunenfeld, B. and Karasik, A. (1994). Hyperinsulinemia induces a reversible impairment in insulin receptor function leading to diabetes in the sand rat model of non-insulin-dependent diabetes mellitus, *Proc. Natl. Acad. Sci. USA*, **91**, 1853-1857.
[18] Shafrir, E. and Ziv, E. (1998). Cellular mechanism of nutritionally induced insulin resistance: The desert rodent *Psammomys obesus* and other animals in which insulin resistance leads to a detrimental outcome, *J. Basic Clin. Physiol. Pharm.*, **9**, 347-385.
[19] Gadot, M., Leibowitz, G., Shafrir, E., Cerasi, E., Gross, D. J. and Kaiser, N. (1994). Hyperproinsulinemia and insulin deficiency in the diabetic *Psammomys obesus*, *Endocrinology*, **135**, 610-616.
[20] Bar-On, H., Ben-Sasson, R., Ziv, E., Arar, N. and Shafrir, E. (1999). Irreversibility of nutritionally induced NIDDM in *Psammomys obesus* is related to beta-cell apoptosis, *Pancreas*, **18**, 259-265.
[21] Habito, R. C., Barnett, M., Yamamoto, A., Cameron-Smith, D., O'Dea, K., Zimmet, P. and Collier, G. R. (1995). Basal glucose turnover in *Psammomys obesus*: An animal model of Type 2 (non-insulin-dependent) diabetes mellitus, *Acta Diabetologica*, **32**, 187-192.
[22] Zimmet, P., Whitehouse, S. and Kiss, J. (1979). Ethnic variability in the plasma insulin response to oral glucose in Polynesian and Micronesian subjects, *Diabetes*, **28**, 624-628.
[23] DeFronzo, R. A. (1988). The triumvirate B-cell, muscle and liver: A collusion responsible for NIDDM, *Diabetes*, **37**, 667-688.
[24] Waaler, H. T., Height, weight and mortality: The Norwegian experience, *Acta Med. Scand.*, **679**, 1-56.
[25] Hoffmans, M. D. A. F., Kromhout, D. and de Lezenne Coulander, C. (1988). The impact of body mass index of 78,612 18-year-old Dutch men on 32-year mortality from all causes, *J. Clin. Epidemiol.*, **41**, 749-756.
[26] Bray, G. A. (1995). Commentary on classics in obesity: Life insurance and overweight, *Obes Res.*, **3**, 97-99.
[27] Despres, J. P., Moorjani, S., Lupien, P. J., Tremblay, A., Nadeau, A. and Bouchard, C. (1990). Regional distribution of body fat, plasma lipoproteins, and cardiovascular disease, *Arteriosclerosis*, **10**, 497-511.
[28] Bjorntop, P. (1991). Metabolic complications of body fat distribution, *Diabetes Care*, **14**, 1132-1143.
[29] Walder, K., Willet, M., Zimmet, P. and Collier, G. R. (1997). *Ob* (obese) gene expression and leptin levels in *Psammomys obesus*, *Biochim. Biophys. Acta*, **1354**, 272-278.

